# Supplementary material for: Proton-Pump Inhibitors and Hypomagnesaemia in Kidney Transplant Recipients
Source: J Clin Med. 2019 Dec 6;8(12):2162. doi: 10.3390/jcm8122162 (PMC6947083; doi:10.3390/jcm8122162)
Supplement: Supplementary file 1 [file jcm-08-02162-s001.pdf]

## Supplemental Tables

**Table S1.** Types and daily dosages of proton-pump inhibitors used by 389 kidney transplant recipients.

| Use of PPIs, daily dose in mg | N (%) |
|---|---|
| Omeprazole | 340 (87.4) |
| 10 mg | 2 (0.5) |
| 20 mg | 231 (59.4) |
| 40 mg | 94 (24.2) |
| 60 mg | 2 (0.5) |
| 80 mg | 11 (2.8) |
| Esomeprazole | 30 (7.7) |
| 20 mg | 9 (2.3) |
| 40 mg | 16 (4.1) |
| 80 mg | 4 (1.0) |
| 160 mg | 1 (0.3) |
| Pantoprazole | 16 (4.1) |
| 10 mg | 1 (0.3) |
| 20 mg | 7 (1.8) |
| 40 mg | 6 (1.5) |
| 80 mg | 2 (0.5) |
| Rabeprazole | 3 (0.8) |
| 10 mg | 2 (0.5) |
| 20 mg | 1 (0.3) |

Data are presented as number and percentage of total.

**Table S2.** Logistic regression analyses investigating the association of PPI use with hypomagnesaemia in 671 kidney transplant recipients (H2RA users excluded).

| | **Hypomagnesaemia** | | |
|---|---|---|---|
| N= 671 | Odds ratio | 95% CI | *P* |
| Crude model | 2.30 | 1.53 – 3.46 | <0.001 |
| Adjusted model | 2.17 | 1.29 – 3.67 | 0.004 |

Multivariable analyses were adjusted for age, sex, BMI, eGFR, proteinuria, time since transplantation, alcohol use, diabetes, history of CV disease, loop diuretics, thiazide diuretics, tacrolimus use, cyclosporine use, MMF use, dietary magnesium intake. Abbreviations: CI, confidence interval.

**Table S3.** Baseline characteristics of 689 RTR with and without hypomagnesaemia.

| **Characteristics** | **RTR with hypomagnesaemia** | **RTR without hypomagnesaemia** | ***P*** |
|---|---|---|---|
| Number of subjects , n (%) | 145 (21%) | 544 (79%) | n/a |
| Demographics | | | |
| Age, years | 52 ± 12 | 53 ± 13 | 0.4 |
| Men, n (%) | 93 (64.1) | 302 (55.5) | 0.06 |
| BMI, kg/m$^2$ | 26.8 ± 5.0 | 26.6 ± 4.7 | 0.6 |
| Diabetes Mellitus, n (%) | 38 (26.2) | 127 (23.3) | 0.5 |
| History of CV disease, n (%) | 73 (50.3) | 201 (36.9) | 0.003 |
| Time since transplantation, years | 4.1 [1.0 – 10.0] | 6.0 [2.4 – 13.0] | <0.001 |
| Lifestyle parameters | | | |
| Current smoker, n (%) | 20 (14.8) | 64 (12.6) | 0.5 |
| Alcohol consumer, n (%) | 80 (62.0) | 356 (72.5) | 0.02 |
| Magnesium intake, mg/d | 333.9 ± 92.6 | 328.9 ± 87.7 | 0.6 |
| Renal function parameters | | | |
| eGFR, ml · min$^{-1}$ · 1.73 m$^{-2}$ | 51.6 ± 20.4 | 52.5 ± 20.1 | 0.6 |
| Serum creatinine, µmol/L | 132.0 [103.0 – 168.8] | 122.0 [99.0 – 158.0] | 0.1 |
| Proteinuria (≥0.5 g/24h), n (%) | 33 (22.8) | 124 (22.9) | 1.0 |
| Laboratory parameters | | | |
| Plasma magnesium, mmol/L | 0.63 ± 0.07 | 0.81 ± 0.08 | <0.001 |
| 24h-urinary magnesium excretion, mmol/24h | 2.91 [1.92 – 3.96] | 3.32 [2.38 – 4.48] | 0.001 |
| Serum potassium, mmol/L | 4.0 ± 0.53 | 3.98 ± 0.45 | 0.4 |
| Serum calcium, mmol/L | 2.38 ± 0.15 | 2.41 ± 0.15 | 0.02 |
| PTH, pmol/L | 10.7 [5.9 – 16.8] | 8.8 [6.0 – 14.0] | 0.2 |
| Glucose, mmol/L | 5.4 [4.9 – 6.2] | 5.2 [4.7 – 6.0] | 0.01 |
| HbA1c, mmol/mol | 41.0 [37.7 – 44.3] | 39.9 [36.6 – 44.3] | 0.03 |
| Medication use | | | |
| Proton-pump inhibitors, n (%) | 102 (70.3) | 287 (52.8) | <0.001 |
| Mycophenolate mofetil, n (%) | 103 (71.0) | 346 (64.2) | 0.1 |
| Tacrolimus, n (%) | 58 (40.0) | 66 (12.1) | <0.001 |
| Cyclosporine, n (%) | 65 (44.8) | 207 (38.1) | 0.1 |
| Sirolimus, n (%) | 0 (0) | 13 (2.5) | 0.08 |
| Prednisolone, n (%) | 142 (97.9) | 540 (99.3) | 0.2 |
| Loop diuretics, n (%) | 28 (19.3) | 132 (24.3) | 0.2 |
| Thiazide diuretics, n (%) | 40 (27.6) | 80 (14.7) | <0.001 |
| H2-receptor antagonists, n (%) | 5 (3.4) | 13 (2.4) | 0.6 |
| Combination therapy | | | |
| MMF + Tac + pred, n (%) | 39 (26.9) | 39 (7.2) | <0.001 |
| MMF + cyclosporine + pred, n (%) | 43 (29.7) | 132 (24.3) | 0.2 |
| MMF + Tac, n (%) | 41 (28.3) | 40 (7.4) | <0.001 |
| MMF + Pred, n (%) | 100 (69.0) | 347 (63.8) | 0.3 |
| MMF + Cyclo, n (%) | 44 (30.3) | 133 (24.4) | 0.2 |
| Cylco + Pred, n (%) | 64 (44.1) | 205 (37.7) | 0.2 |
| Tac + Pred, n (%) | 56 (38.6) | 64 (11.8) | <0.001 |

Data are presented as mean ± SD, median with interquartile ranges (IQR) or number with percentages (%). Abbreviations: BMI, body mass index; eGFR, estimated glomerular filtration rate; HbA1c, hemoglobin A1c; PTH, Parathyroid hormone; MMF, mycophenolate mofetil; Tac, tacrolimus; Pred, prednisolone.
